# In Vivo Evaluation of Sgc8-c Aptamer as a Molecular Imaging Probe for Colon Cancer in a Mouse Xenograft Model

**DOI:** 10.3390/ijms23052466

**Published:** 2022-02-23

**Authors:** Ana Paula Arévalo, Romina Castelli, Manuel Ibarra, Martina Crispo, Victoria Calzada

**Affiliations:** 1 Laboratory Animals Biotechnology Unit, Institut Pasteur de Montevideo, Montevideo 11400, Uruguay; aparevalo@pasteur.edu.uy; 2 Área de Radiofarmacia, Centro de Investigaciones Nucleares, Facultad de Ciencias, Universidad de la Republica, Montevideo 11400, Uruguay; romi.castelli@gmail.com; 3 Department of Pharmaceutical Sciences, Faculty of Chemistry, Universidad de la Republica, Montevideo 11800, Uruguay; mi.cebiobe@gmail.com

**Keywords:** PTK7, colon adenocarcinoma, LS174T, molecular imaging, theranostics

## Abstract

Recent biotechnological applications in the field of clinical oncology led to the identification of new biomarkers as molecular targets of cancer, and to broad developments in the field of personalized medicine. Aptamers are oligonucleotides (ssDNA or RNA) that are selected to specifically recognize a molecular target with high affinity and specificity. Based on this, new horizons for their use as molecular imaging probes are being explored. The objective of this work was to evaluate the Sgc8-c aptamer conjugated with Alexa Fluor 647 fluorophore as an imaging probe in a colon tumor xenograft mouse model, with potential application in molecular imaging. In this study, the LS174T cell line was used to induce colorectal adenocarcinoma in nude mice. After confirmation of PTK7 overexpression by immunohistochemistry, in vivo studies were performed. Pharmacokinetic, in vivo and ex vivo biodistribution imaging, and a competition assay were evaluated by fluorescence imaging. In vivo visualization of the probe in the tumors was assessed two hours after aptamer probe administration, exhibiting excellent tumor-to-background ratios in biodistribution studies and high specificity in the competition test. Our results demonstrated the functionality of Scg8-c as an imaging probe for colon cancer, with potential clinical applications.

## 1. Introduction

According to a World Health Organization report from 2020, cancer is one of the principal causes of death, with 10 million deaths in 2021 [1]. The five types of cancer that cause the highest number of deaths in 2020 were lung (1.8 million), colorectal (935,000), liver (830,000), gastric (769,000), and breast (685,000). Over 30–50% of cancers can be prevented, reduced, or mitigated by avoiding external risk factors or by adopting public health measures aimed for prevention, such as adequate screening and diagnoses. Early diagnosis and competent controls increase survival, reducing global morbidity [1,2]. Recent advances in imaging technology have positioned this kind of research at the forefront of scientific studies of the diagnosis and evaluation of diverse pathologies. These techniques allow real-time monitoring and generate a large amount of information, not only on the focus of the study, but on the biological environment [3]. The term molecular imaging can be broadly defined as the in vivo characterization and measurement of biologic processes at the cellular and molecular levels [4]. The development of novel agents, signal amplification strategies, and improved imaging technologies is the focus for this technological tool. Specific imaging of biomarkers allows earlier detection and characterization of a given disease, earlier and direct molecular assessment of treatment effects, and a more fundamental understanding of the disease process [4]. Imaging modalities such as ultrasound (US), X-ray (Rx), magnetic resonance imaging (MRI), computed tomography (CT), positron emission tomography (PET), single-photon emission tomography (SPECT), and optical imaging (OI) allow the creation of in vivo images, providing information additional to the classic diagnosis, monitoring, and prognosis in oncological pathologies, and can be used to guide different medical procedures in situ [5,6,7]. The potential advantages of using a fluorophore-labeled aptamer over other agents for tumor imaging are its availability as a kit, safety, and ease of use. Furthermore, optical imaging windows exist in the 600–1000 nm area (near-infrared (NIR)) of the electromagnetic spectrum where light absorption of biological molecules is minimal. In addition, light scattering and autofluorescence are lower in the NIR, enabling higher signal-to-background ratios [8].

Molecular imaging probes present different characteristics depending on the purpose of the study. For many years, the use of antibodies was a reference in this field, with some limitations in terms of half-life. Recently, aptamers are being more explored in molecular imaging. Aptamers are oligonucleotides (ssDNA or RNA) that are selected to recognize a molecular target with high affinity and specificity. Several aptamers have been developed for biomedical applications, including molecular imaging, diagnostics, therapies, drug administration, and clinical procedures [9,10,11,12]. The Food and Drug Administration (FDA) has approved aptamers as therapeutic agents, with several clinical trials currently underway [9,10,11,12]. Furthermore, their low molecular weight (~15 kDa) and interesting physicochemical properties promote rapid tissue penetration and fast clearance, achieving high target/nontarget ratios, indicating their potential as good imaging agents in cancer [13,14]. Sgc8-c is a well-characterized hairpin DNA aptamer (41 bases) that recognizes the tyrosine kinase 7 protein (PTK7) with *K*d = 0.78 nM [15,16,17]. This receptor is overexpressed in multiple cancers and has been proposed as a prognostic marker in several tumors [18,19,20,21]. Previously, the Sgc8-c aptamer was explored in vivo by our group as a molecular imaging probe, achieving excellent results [22,23,24,25,26,27]. 

The objective of this work was to evaluate the performance of Sgc8-c aptamer linked to Alexa Fluor 647 in a mouse xenograft model of colon adenocarcinoma, as an in vivo imaging probe.

## 2. Results

### 2.1. Probe Stability

The stability of Sgc8-c-Alexa647 was monitored using SEC-HPLC at ʎ = 260 and 650 nm. After 40 min, and 1 and 2 h of incubation with mouse serum, the retention time did not change and there was no additional evidence of fragmentation of the probe, with a retention time (t_R_) of 7.2 min. However, a small shoulder at t_R_ = 7.8 min and ʎ = 650 nm was observed after 24 h of incubation, corresponding to smaller species, quantified in less than 20% of the area under the curve (AUC). A small oligonucleotide control of MW = 5537 Da was eluted at t_R_ = 8.7 min (see Supplementary Material). Thus, these experimental findings support the matrix stability of Sgc8-c-Alexa647 in serum for 2 h at 37 °C under the SE-HPLC conditions employed, following the absence of significant degradation in that period (Figure S1).

### 2.2. Tumor Induction

Xenotransplanted mice showed palpable tumors 9 days post-injection and reached a size of 200–500 mm^3^ 20 days post-injection. Animals were daily monitored and no welfare concerns were registered. 

### 2.3. Pharmacokinetics

Four animals presented quantifiable aptamer levels in blood up to 60 min post-administration, while only one mouse presented quantifiable levels up to 240 min post-administration. 

Observations were more accurately described by a two-compartment model with first-order rate constants for intercompartment aptamer distribution and elimination from the central compartment. The BICc of this model was 194 versus 264 for the one-compartment model. Data were analyzed using log-transformation and the residual error was described using an exponential model, with a mean magnitude (relative standard error) of 0.27 (15.2%). Mice body weight was included in each disposition parameter accounting for the size effect on aptamer distribution and elimination according to an allometric scaling model with a coefficient of 0.75 for elimination and distribution clearances, and 1 for the volume of distribution of the central and the peripheral compartment. Estimations of the mean (relative standard error) pharmacokinetic parameters for 25 g mice were 81.77 mL (20.9%) and 182.2 mL (48.7%) for central and peripheral distribution volume, respectively. Elimination clearance was 3.29 mL/min (15.1%) and distribution clearance was 1.78 mL/min (20.8%). Between-animal variability was included for the elimination clearance, resulting in 22% (38.5%), expressed as coefficient of variability (relative standard error). Individual fits, visual predictive check, and other goodness-of-fit plots are included as Supplementary Material (Supplementary Material Figures S2–S4).

### 2.4. Biodistribution 

**Figure 1:**
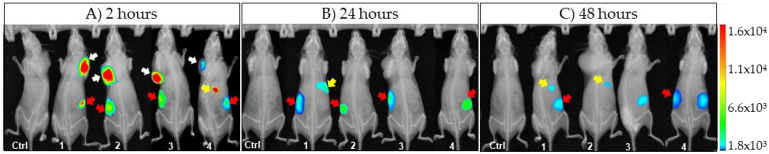
Images acquired post-injection of Sgc8-c-Alexa647: arrows indicate fluorescent signals detected in vivo (white = tumors; yellow = liver and gallbladder; red = kidneys). Aptamer probe injected mice (1, 2, 3 and 4) and PBS injected mice (Ctrl).

Two hours post Sgc8-c-Alexa647 injection, images of whole mice were acquired and tumors were clearly visualized, as shown in Figure 1A. In vivo fluorescence signal decreased in tumors after 24 h (Figure 1B,C), corroborating the early clearance of the aptamer as observed in the pharmacokinetic study.

**Figure 2:**
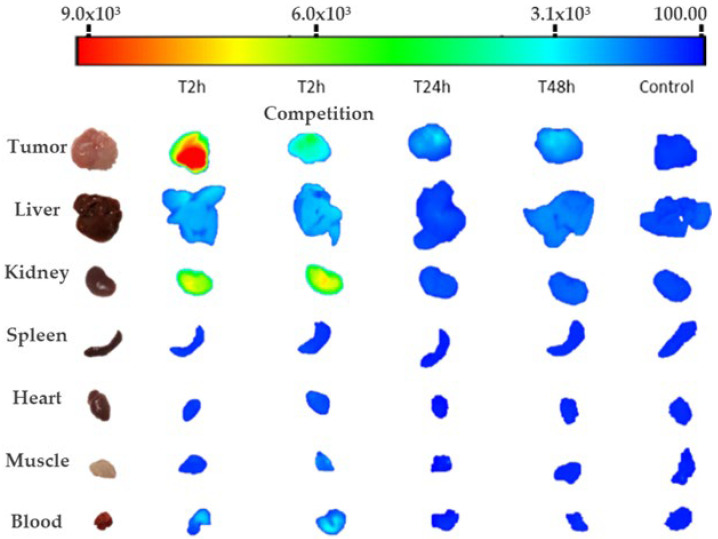
Representative image of quantified fluorescence expressed in principal organs for every ex vivo assay performed.

**Figure 3:**
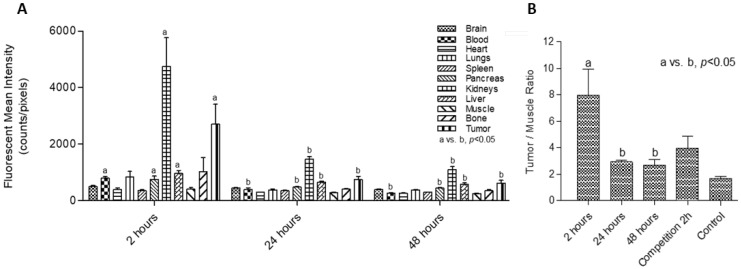
(**A**) Biodistribution assay: mean fluorescence intensity quantified per organ per group at each timepoint. Different letters (a,b) indicate significant differences (*p* <  0.05) between fluorescence in the same organ at 2 h versus 24 and 48 h. (**B**) Tumor/muscle ratio per group. Different letters (a,b) indicate significant differences (*p* <  0.05) between ratios at 2 h versus 24 and 48 h.

To visualize fluorescent signal from all internal organs, ex vivo images were acquired after each in vivo acquisition (Figure 2). As expected, a high signal was detected in tumors for all evaluated times. Additionally, kidneys showed a high signal two hours after probe injection and fluorescence detected over time corresponding with high clearance of the probe. A decrease in the fluorescence signal in tumors was observed after 24 h (Figure 2 and Figure 3).

The highest ex vivo fluorescence values in the excretion routes were obtained from the liver and kidneys two hours after probe injection. Higher values in the liver than in other organs were detected, suggesting early hepatobiliary metabolization (Figure 3A). Statistical differences at 2 h in comparison to 24 and 48 h were observed in the following organs and fluid: liver, kidneys, pancreas, and blood (*p* < 0.05). There was also a statistical difference between tumor uptake values obtained at 2 h with respect to 24 and 48 h (*p* < 0.05; Figure 3). Finally, the tumor/muscle values (Figure 3B) showed that the tumor-to-background ratio was highest at 2 h post-injection of the probe, as we observed in in vivo images.

### 2.5. Competition Assay

**Figure 4:**
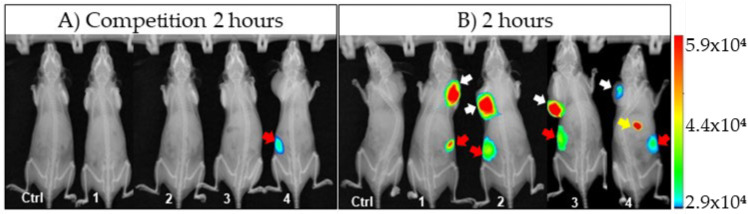
Comparative images acquired two hours post-injection of: (**A**) Sgc8-c-NH_2_ + Sgc8-c-Alexa647; (**B**) Sgc8-c-Alexa647 alone. Arrows indicate fluorescent signals detected in vivo (white = tumors; yellow = liver and gallbladder; red = kidneys). Aptamer-probe-injected mice (1, 2, 3 and 4) and PBS-injected mice (Ctrl).

In vivo images obtained 2 h post-injection of the unlabeled aptamer Sgc8-c-NH_2_ and Sgc8-c-Alexa647 showed renal uptake in one mouse, with no signal from other organs (Figure 4A). For ex vivo images, higher fluorescent signals were observed in the metabolization and main elimination pathways (liver and kidneys), showing the highest measure in kidneys from animal #4, in concordance with the in vivo images obtained. Although an increase in the average liver measure was detected, a similar uptake comparable to the 2 h measures was obtained from the rest of the organs. The tumor uptake average was lower than that obtained at 2 h (1325 vs. 3273 counts/pixels, respectively, showing similar results to those obtained at 24 and 48 h post-injection with Sgc8-c-Alexa647 (850 and 690 counts/pixel, respectively; Figure 2, Table S1). 

### 2.6. Histological and Immunohistochemical Analysis 

**Figure 5:**
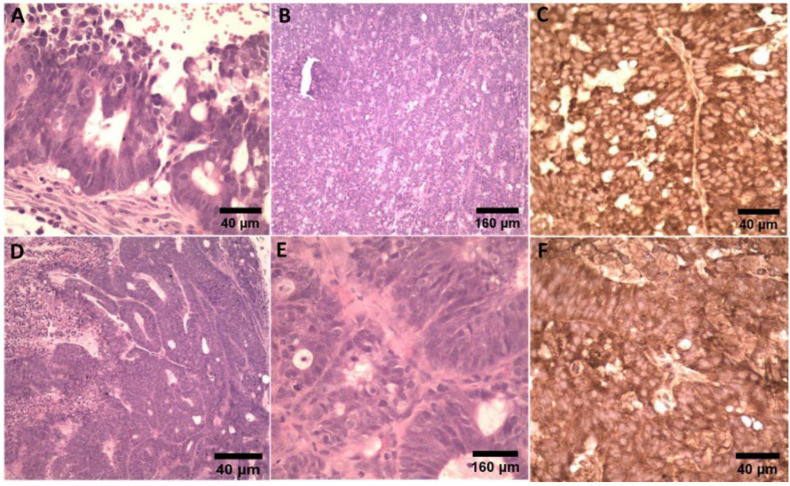
Micrographs of hematoxylin and eosin (H&E), HER2, and PTK7 IHC staining of harvested LS174T tumor tissue. LS174T tumors were PTK7-positive and HER2-negative. (**A**–**C**) Tumor #1 showed minimal incomplete membrane expression for HER2 (score 1+) and diffuse cytoplasmic positivity for PTK7. (**D**–**F**) For tumor #2, HER2 expression was not observed (score 0) and diffuse cytoplasmic positivity for PTK7.

The histology results from tumors induced with LS174T samples confirmed sections of malignant epithelial neoplasia with necrotic and apoptotic regions. Stromal desmoplastic reaction was also observed. The immunohistochemistry assay determined a carcinoma that exhibited a diffuse and strong cytoplasmic staining for PTK7 that was negative for HER2 (score 0 to 1+) (Figure 5).

## 3. Discussion

Research and development of new tools and strategies for early cancer diagnosis, prevention, and personalized therapy are critical to reduce morbidity and mortality in the future. Colorectal cancer was the third most common malignancy (1.9 million incidence) and the second deadliest cancer (0.9 million deaths) worldwide in 2020 and adenocarcinomas account for about 96% of colorectal cancers [28]. The common diagnostic for this type of pathology is invasive: colonoscopies, biopsy, or biomarker testing of the tumor with a battery of imaging techniques used for detection, helping with related procedures such as sampling, guided surgeries, or monitoring therapy [29,30]. Samples can reveal several biomarkers that have been described to molecular subtypes, providing important information about prognosis and the possibility of specific target therapies. Molecular imaging based in NIR fluorescence can effectively identify biomarkers and enhance image contrast, improving the early detection of neoplasia compared to conventional wide-light screening. Furthermore, some NIR imaging probes have received approval from the Food and Drug Administration (FDA) for clinical use [8]. Thus, new fluorescents probes are being tested for different diagnostic and theranostic procedures [31,32,33,34,35]. Fluorescence-guided surgery can provide real-time imaging during surgery, and it is much easier and reliable to perform [30]. PTK7 is overexpressed in colon adenocarcinoma [18,36] and is a promising biomarker od aggressiveness and metastasis [37]. Furthermore, antibody–drug conjugate targeting protein tyrosine kinase 7 demonstrated therapeutic activity in a human study with advanced, moderate or high PTK7 expression, solid tumors [38].

As previously reported, Sgc8-c aptamer specifically recognizes PTK7 and it has been functionalized as a molecular imaging probe [18,22,23,24,25,26,27,39]. In this study, we evaluated an aptamer-based fluorescent molecular imaging probe in a mice xenograft colon adenocarcinoma model. This type of murine model is widely used to replicate carcinogenesis, for the evaluation of therapeutics strategies, and for the discovery of sensitive and specific biomarkers [40]. Here, the estimation of the pharmacokinetic parameters showed that the central and peripheral distribution volumes were 81.77 and 182.2 mL, respectively. The obtained values indicated that there was a rapid and extensive distribution of the aptamer to the rest of the tissues of the organism compared to antibodies or fragments, which showed lower values [41,42]. The total estimated elimination clearance was 3.29 mL/min. This value is higher than liver blood flow in mice (1.8 mL/min), suggesting other elimination pathways such as renal or intestinal pathways. Aptamer exposure was proportional to the given dose within the experimented dose range. Estimated disposition half-lives were 10.52 and 115.8 min, indicating an initial rapid distribution and an important accumulation in peripheral tissues, as previously reported [26]. Although the probe cleavage in serum during the monitored time can be neglected, one limitation of pharmacokinetic analysis that must be acknowledged is the potential contribution of aptamer metabolites to the fluorescent signal. Thus, metabolite disposition could have altered the estimated pharmacokinetic parameters. 

Fluorescence uptake could be detected mostly in tumors for all image determinations. Because the tumor-to-background ratio should be as high as possible for imaging purposes [31,43], we evaluated the signal at different time points and found that the highest signal and tumor/muscle ratio were obtained 2 h post-injection of Sgc8-c-Alexa647. Thus, of the evaluated conditions, 2 h was the best time point for in vivo or ex vivo tumor visualization. The detection limits and optimal probe doses should be further evaluated. 

Additionally, the fluorescence signal was recorded from the liver and kidneys. The elevated kidney signal could have been the result of the elimination of the fluorescent probe or its metabolites through this metabolic pathway. In addition, the specificity of the aptamer was evaluated in vivo though a competition assay [44,45]. Fluorescence values were observed in kidneys and liver, as expected, because the unlabeled aptamer previously occupied the receptors, leaving less binding availability for the labeled Sgc8-c-Alexa647, evidencing their specificity.

In conclusion, our results demonstrated the functionality of Scg8-c-Alexa647 as a probe for molecular imaging in a xenograft colon adenocarcinoma mouse model. Although further studies should be conducted to gather more information, these data suggest that Scg8-c is a promising tool for theranostics and guided-clinical procedures in human cancer. 

## 4. Materials and Methods

### 4.1. Sgc8-c Labeling

Sgc8-c aptamer (MW = 12.6 kDa) was tagged with Alexa Fluor 647 NHS following our established procedure [22,27]. Briefly, 5′-aminohexyl-modified Sgc8-c aptamer (41 nt) (Sgc8-c-NH_2_, 5′-/AM/ATC TAA CTG CTG CGC CGC CGG GAA AAT ACT GTA CGG TTA GA-3′) was purchased from IDT (Integrated DNA Technologies, Coralville, IA, USA). Alexa Fluor 647- succinimidyl ester (Alexa647-NHS) was purchased from Thermo Fisher Scientific (A-20006, Thermo Fisher Scientific, Waltham, MA, USA). To prepare the fluorescent probe (Sgc8-c-Alexa647), a solution of Sgc8-c-NH_2_ (1 mg) was dissolved in Milli-Q purified water (300 µL, pH 8.5, adjusted with 0.1 M aqueous NaHCO_3_), and Alexa647-NHS (1:2 molar ratio) dissolved in dry dimethylsulfoxide (DMSO) (10 µL) was added. The mixture was stirred at room temperature for 30 min in the dark. We washed 5′-/Alexa647AM/ATC TAA CTG CTG CGC CGC CGG GAA AAT ACT GTA CGG TTA GA-3′ (Sgc8-c-Alexa647) in a microcon^®^ Centrifugal Filters 10 kDa cut-off (MerckMillipore, Darmstadt, Germany), and then purified and analyzed it by reverse-phase high-performance liquid chromatography (RP-HPLC). The Sgc8-c-Alexa647 bioconjugated 100% pure was washed in water with a centrifugal filter and finally freeze-dried and stored at −20 °C until use. The stability of the Sgc8-c-Alexa647 was analyzed by the incubation of 40 µg of aptamer in fresh mouse serum at 37 °C. The integrity of the probe was followed at ʎ = 260 and 650 nm over 24 h after incubation by size-exclusion chromatography (SEC) with isocratic elution of phosphate buffer (0.05 M, pH = 7, and flow = 1 mL/min) in a Protein-Pak^TM^ 300 SW (7.5 × 300 mm) size-exclusion HPLC column (Waters Corporation, Milford, MA, USA).

### 4.2. Animals

Twenty-six nu/J female mice (Jax Stock # 002019) aged 8 to 12 weeks, reared at the specific pathogen-free (SPF) animal facility of Laboratory Animal Biotechnology Unit (UBAL), were used. Mice were housed in groups of five in individually ventilated cages (AL2, ALESCO, Sao Paulo, SP, Brazil) in a controlled environment with the following standardized parameters: 14/10 light/dark cycle, 21 ± 2 °C room temperature, and 50–70% of humidity. They were fed with commercial food (5K67, LabDiet, St. Louis, MO, USA) ad libitum and had free access to filtered and autoclaved water. One week prior to the experiments, mice were transported to the experimental area for acclimatization, maintaining the same housing conditions and standardized parameters. For the studies that involved in vivo fluorescence images acquisition, the animals were fasted 12 h prior to the study in order to reduce possible interference in the spectrum range obtained both for chlorophyll (present in the food) and the fluorophore used. Animal care was carried out in compliance with National Experimentation Law No. 18.611 and the UBAL’s Guide for the care of small rodents. The Institut Pasteur de Montevideo Animal Use Ethics Commission approved all animals’ procedures (# 009/12 and 014/19).

### 4.3. Tumor Induction

The LS174T (ATCC^®^ CL188™) cell line from a human colon adenocarcinoma was used to induce tumors in mice. For culture, the cell line was kept in 75 cm^3^ culture bottles in an incubator at 37 °C in a humid atmosphere of 5% CO_2_, in Dulbecco’s modified eagle medium (DMEM, Gibco™, Life Technologies, Grand Island, NE, USA) supplemented with 10% fetal bovine serum (SFB, Gibco™, Life Technologies, Grand Island, NE, USA). When an 80–90% confluence monolayer was established, cells were recovered by a scraper in PBS (Sigma Aldrich, St. Louis, MO, USA) for mouse injection. Sixteen 8–12-week old female nu/J mice were subcutaneously injected with 1 × 10^6^ cells resuspended in 100 µL of sterile PBS into the dorsum-cervical area. Tumor growth was monitored weekly with a caliper and the volume was calculated using the formula V = ½ × W^2^ × L [46,47]. 

### 4.4. Pharmacokinetics

For this study, nontumor-induced nu/J mice (*n* = 6) were injected intravenously in the tail with 40 µg of Sgc8-c-Alexa647 in 100 µL of PBS, in bolus. To assess the pharmacokinetic profile, serial blood samples were measured. Complete blood samples (30 µL) were taken at 0 (pre-injection), 15, 30, 60, 90, and 120 min and at 4, 8, 24, and 48 h post-injection by submandibular puncture. Blood was mixed with 3 µL of anticoagulant (W EDTA 3K, Wiener Lab Group, Montevideo, Uruguay) and loaded on black polystyrene microplates (Thermo Scientific™, Waltham, MA, USA). for fluorescence quantification. For fluorescence measurement, all samples were quantified using an excitation 650 nm and emission 700 nm filters at different times in an In-Vivo Xtreme II™ (Bruker Daltonik Gmbh, Billerica, MA, USA) and analyzed using V.7.5.3.22464 MolecularImaging software (Bruker Daltonik Gmbh, Billerica, MA, USA).

Pharmacokinetic analysis to calculate the distribution and elimination parameters was performed. The model-building process was driven by several diagnostic metrics and graphics. The corrected Bayesian information criterion (BICc) computed from the estimated log-likelihood was used to optimize model parsimony, assessing the tradeoff between data fit and model complexity. Basic goodness-of-fit plots included observations versus individual and population predictions, residuals versus time and versus the MTX concentration, and the distribution of residual error. In addition, simulation-based diagnostics, visual predictive check (VPC) and normalized prediction distribution errors (NPDE), were also considered. 

### 4.5. Biodistribution

To study aptamer biodistribution, 12 mice with tumors were intravenously administered in the tail with 40 µg Sgc8-c-Alexa647 in 100 µL of PBS. At 2, 24, and 48 h post-injection (*n* = 4), mice were anaesthetized with isoflurane and in vivo images were acquired using the same parameters as described above. Immediately, mice were sacrificed by cervical dislocation and organ dissection was performed to measure ex vivo fluorescence. One control mouse with only PBS injection was included in each group of study. To improve probe uptake visualization per organ, the stomach, intestine, and gallbladder were not included in the images acquisition since they present high autofluorescence [48]. Mean fluorescence intensity levels from organs were evaluated, as well as the tumor-to-background ratios (TBRs) using tumor/muscle values [49].

### 4.6. Competition Assay

Aptamer specificity was determined through a competition assay. To evaluate the probe’s specific binding to the target, four mice with tumors were intravenously administered in the tail with an injection of unlabeled aptamer in excess (200 µg Sgc8-c-NH_2_). After 30 min, the labeled aptamer (40 µg Sgc8-c-Alexa647) was injected. Two hours’ post-injection, mice were anaesthetized with isoflurane and in vivo images were acquired using the same parameters as described above. Immediately, mice were sacrificed by cervical dislocation and organ dissection was performed to measure ex vivo fluorescence.

### 4.7. Histological and Immunohistochemical Analysis

Two samples of tumors were evaluated by histology and immunohistochemistry using HER2 (anti-ErbB2/HER2 antibody (SP3) (ab16662), ABCAM, Boston, MA, USA) as a negative marker and PTK7 (PA5-97262, catalog #PA5-97262 Invitrogen, Thermo Fisher Scientific, Waltham, MA, USA) as a positive marker. To assess PTK7 overexpression in this xenograft colon adenocarcinoma model, the tumors were fixed immediately after necropsy in 10% neutral buffered formalin (pH 7.4) for further processing. For evaluation, they were embedded in paraffin and sectioned in 4 µm sections and stained with hematoxylin–eosin (H&E). Immunohistochemistry (IHC) as performed according to Tian and Damelin et al. [18,50].

### 4.8. Statistical Analysis

The pharmacokinetic analysis of measured fluorescence per sample volume was performed by nonlinear mixed effects modeling (NLME) using MonolixSuite 2020R2 (Lixoft SAS, Antony, France). Statistical analysis using mixed generalized linear models from InfoStat [51] was performed for biodistribution.

## Data Availability

All data are available within the article and in Supplementary Materials.

## Supplementary Materials

The following are available online at https://www.mdpi.com/article/10.3390/ijms23052466/s1.
