# Supplementary material for: In Vivo Evaluation of Sgc8-c Aptamer as a Molecular Imaging Probe for Colon Cancer in a Mouse Xenograft Model
Source: Int J Mol Sci. 2022 Feb 23;23(5):2466. doi: 10.3390/ijms23052466 (PMC8910571; doi:10.3390/ijms23052466)

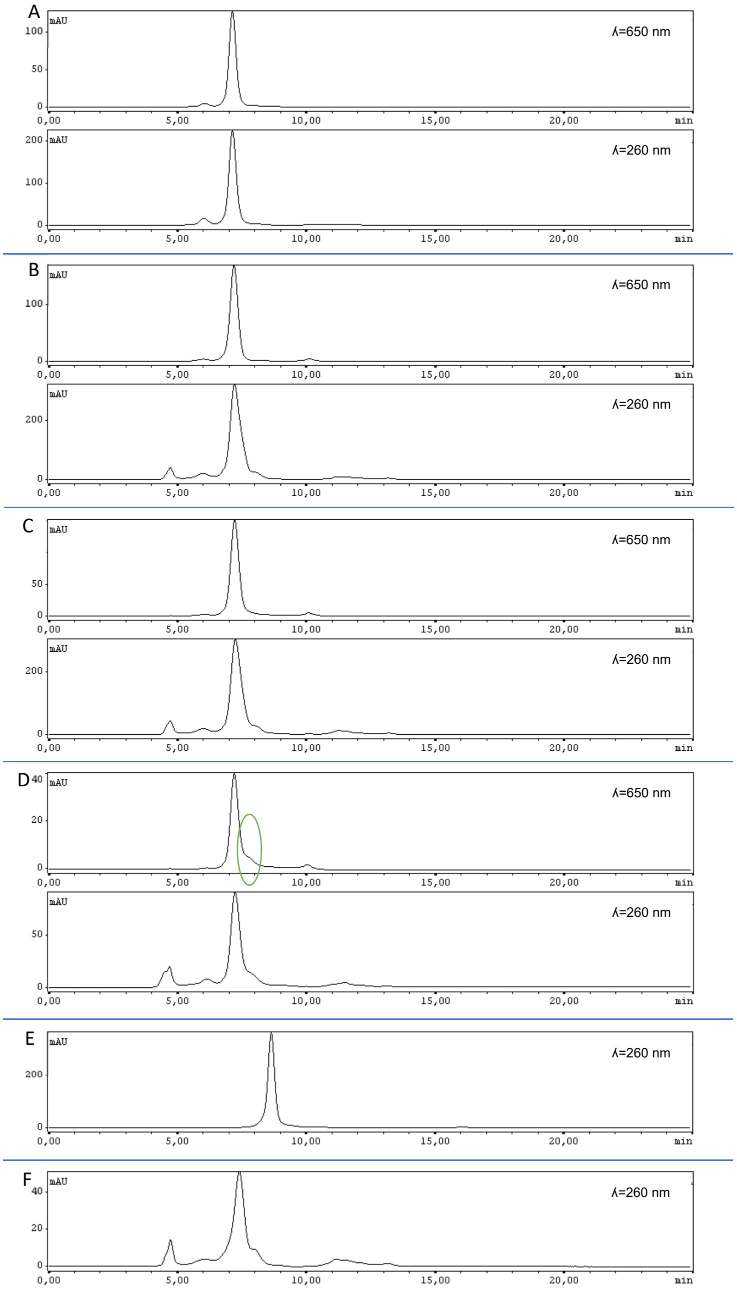

Figure S1. SE-HPLC stability evaluation of Sgc8-c-Alexa647 in serum. The figure shows 650nm and 260nm chromatograms of Sgc8-c-Alexa647 (A), and 40 min (B), 2 hours (C), and 24 hours (D) of incubation in mouse serum. A small shoulder at t_R=7.8 min in the green circle was observed at 24 hours after incubation. A small oligonucleotide of MW = 5537 Da t_R=8.7 min was used as control of fragmentation (E) and mouse serum (F) was also monitored.

Table S1. Biodistribution: Mean fluorescence intensity values per organ per animal.

Organ	**2 h post injection**							**24 h post injection**						
	**Mean fluorescence intensity** (counts/pixel)							**Mean fluorescence intensity** (counts/pixel)						
	**1**	**2**	**3**	**4**	**Control**	**PROM**	**σ**	**1**	**2**	**3**	**4**	**Control**	**PROM**	**σ**
Brain	504.521	470.531	506.851	606.472	429.5099	522.094	58.65	451.261	435.932	482.252	475.34	399.351	461.196	21.4508
Blood	736.774	638.391	818.778	979.854	784.8681	793.449	144.5	297.38	488.455	285.057	433.67	476.937	376.141	100.703
Heart	351.319	399.415	392.787	568.631	281.4486	428.038	96.115	324.716	310.015	286.267	299.11	289.799	305.027	16.3248
Lungs	935.309	805.757	472.68	1530.84	417.2441	936.145	441.76	322.634	459.575	290.712	434.099	405.463	376.755	82.6234
Spleen	334.115	360.502	363.922	455.33	299.009	378.467	52.944	313.33	397.467	389.866	346.217	283.405	361.72	39.3787
Pancreas	689.324	781.135	674.382	1164.43	463.1552	827.319	229.65	495.765	484.629	533.39	467.351	466.558	495.284	27.965
Kidneys	6310.59	5850.25	3793.83	6584.13	1215.062	5634.7	1264.1	1837.85	1451.61	1388.42	1396.99	1258.6	1518.72	214.589
Liver	972.153	1089.26	785.663	1274.87	716.8325	1030.49	205.36	764.951	612.234	658.633	650.371	605.542	671.547	65.4663
Muscle	342.08	468.301	412.281	548.926	245.8536	442.897	87.538	269.284	275.663	261.377	348.68	250.719	288.751	40.3777
Bone	646.173	644.386	489.325	3026.47	284.1597	1201.59	1218.8	474.588	440.449	385.359	377.285	332.911	419.42	46.2646
Tumor	4335.58	3395.16	3601	1759.1	426.9561	3272.71	1086.8	793.421	730.171	813.452	1063.29	344.856	850.083	146.502
**Organ**	**48 h post injection**							**Competition Test**						
	**Mean fluorescence intensity** (counts/pixel)							**Mean fluorescence intensity** (counts/pixel)						
	**1**	**2**	**3**	**4**	**Control**	**PROM**	**σ**	**1**	**2**	**3**	**4**	**Control**	**PROM**	**σ**
Brain	392.316	391.762	433.95	408.431	368.1245	406.615	19.795	404.891	507.101	415.232	649.994	512.473	494.304	113.505
Blood	252.736	263.151	251.745	340.785	219.8493	277.104	42.766	364.838	1039.95	584.45	682.707	710.736	667.986	281.337
Heart	267.718	261.813	232.072	292.634	267.7928	263.559	24.88	372.311	482.541	353.884	542.042	277.341	437.695	89.8125
Lungs	368.621	416.318	432.332	336.715	351.7433	388.497	43.863	715.685	879.916	476.447	1531.12	552.635	900.793	451.696
Spleen	291.557	321.784	280.526	315.85	292.5432	302.429	19.602	318.399	367.275	342.857	486.677	290.798	378.802	74.6334
Pancreas	459.149	471.19	429.256	474.445	377.1959	458.51	20.582	708.472	823.182	590.978	923.908	545.138	761.635	143.841
Kidneys	1092.26	948.358	1361.31	1283.12	831.0224	1171.26	186.69	3204.37	5965.06	2648.5	5577.53	1428.37	4348.87	1665.61
Liver	730.343	590.764	624.249	501.598	449.223	611.739	94.506	1054.69	1018.99	767.686	1243.19	657.649	1021.14	195.512
Muscle	249.305	231.046	287.272	276.445	222.4296	261.017	25.578	297.038	639.325	381.806	563.942	243.184	470.528	158.313
Bone	475.751	314.62	328.248	349.613	300.1834	367.058	73.879	365.072	480.728	393.903	444.304	278.136	421.002	51.5516
Tumor	930.212	681.302	549.283	596.612	347.6253	689.352	169.61	1068.65	1717.5	1128.95	1384.68	496.898	1324.94	295.394

Figure S2. Pharmacokinetic: Visual predictive check Sgc8-c blood fluorescence (ROI/µL) vs time (min).

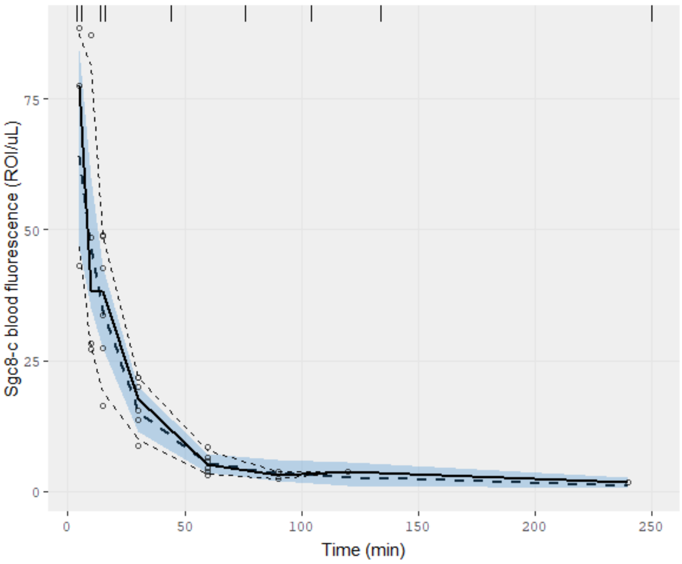

Figure S3. Individual fits for the final model in logarithmic scale. Blue dots correspond to the observations of aptamer in blood. (ROI/µL). The line reflects the model-based pharmacokinetic curve.

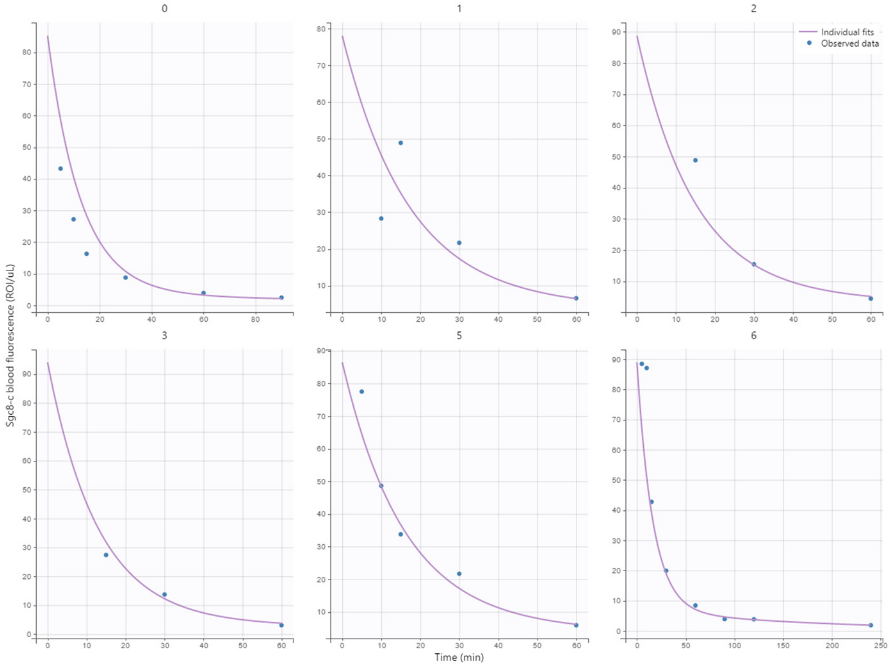

Figure S4. Visual predictive check (VPC) for the final model. Observations are shown as dots. The blue line stands for the median of the observations. Black line represents the model-based predicted median. Overall, the VPC shows a good fit of the data obtained after administration of both formulations.

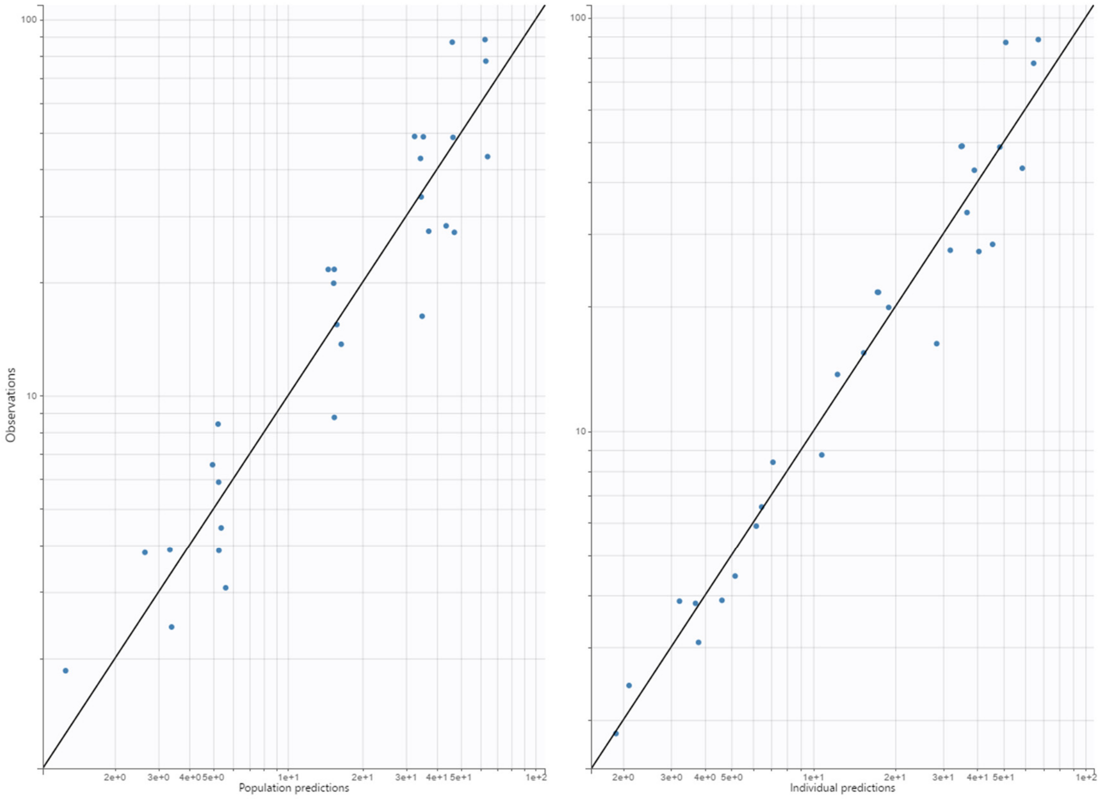

Supplement: Supplementary file 1 [file ijms-23-02466-s001.zip › ijms-1552905-supplementary.pdf]
